# Modeling Physiological Processes That Relate Toxicant Exposure and Bacterial Population Dynamics

**DOI:** 10.1371/journal.pone.0026955

**Published:** 2012-02-06

**Authors:** Tin Klanjscek, Roger M. Nisbet, John H. Priester, Patricia A. Holden

**Affiliations:** 1 Department of Ecology, Evolution and Marine Biology, University of California Santa Barbara, Santa Barbara, California, United States of America; 2 Rudjer Boskovic Institute, Zagreb, Croatia; 3 Bren School of Environmental Science and Management, University of California Santa Barbara, Santa Barbara, California, United States of America; Laurentian University, Canada

## Abstract

Quantifying effects of toxicant exposure on metabolic processes is crucial to predicting microbial growth patterns in different environments. Mechanistic models, such as those based on Dynamic Energy Budget (DEB) theory, can link physiological processes to microbial growth.

Here we expand the DEB framework to include explicit consideration of the role of reactive oxygen species (ROS). Extensions considered are: (i) additional terms in the equation for the “hazard rate” that quantifies mortality risk; (ii) a variable representing environmental degradation; (iii) a mechanistic description of toxic effects linked to increase in ROS production and aging acceleration, and to non-competitive inhibition of transport channels; (iv) a new representation of the “lag time” based on energy required for acclimation. We estimate model parameters using calibrated *Pseudomonas aeruginosa* optical density growth data for seven levels of cadmium exposure. The model reproduces growth patterns for all treatments with a single common parameter set, and bacterial growth for treatments of up to 150 mg(Cd)/L can be predicted reasonably well using parameters estimated from cadmium treatments of 20 mg(Cd)/L and lower.

Our approach is an important step towards connecting levels of biological organization in ecotoxicology. The presented model reveals possible connections between processes that are not obvious from purely empirical considerations, enables validation and hypothesis testing by creating testable predictions, and identifies research required to further develop the theory.

## Introduction

Investigations of the dependence of bacterial growth curves on exposure are often used to consider the ecological importance of toxicants. Although such dependence is an aggregate, population-level measure of all toxic effects, it is a consequence of processes at the cellular or molecular levels.

Toxicants may affect the onset, rate, and extent of bacterial growth curves by directly increasing mortality, impacting nutrient uptake by affecting cross-membrane transport ([1]–[3]), and by disrupting proteins and impeding enzyme function ([4]). In addition, exposure can lead to an increase in energy required for cellular maintenance processes such as protein turnover and defense protein production ([2], [5]–[7]), maintaining ion gradients across cell membranes ([3], [8]), and may affect costs of cell growth in other ways ([7], [9]). Cells can incur additional energy expenses for expelling the toxicant ([10], [11]) and mitigating the effects of toxicant action (DNA/RNA repair, protein repair) ([2], [12]–[15]). Distinguishing among these possibilities using population-level data requires models relating biomolecular-level processes to population dynamics.

Dynamic Energy Budget (DEB) theory provides a comprehensive framework for connecting molecular-level processes to individual physiology and population growth of all organisms ([16]), and has been suggested as a way to connect multiple levels of biological organization necessary for a comprehensive ecotoxicological theory ([17]). DEB models describe energy and material acquisition and accumulation, and consequential commitment of energy to maintenance, growth, and cell division. DEB theory also includes a description of the aging process: excess reactive oxygen species (ROS) cause irreparable damage (e.g. to the cellular DNA). The damaged parts of the cells are called “damage-inducing compounds”: they cause the cell to produce ‘incorrect’ proteins which, in turn, accumulate as damage to the cell and increase the probability of death. To better link the aging process to population-level processes, the DEB theory scales damage-inducing compounds and damage, interpreting the scaled quantities as “aging acceleration” and “hazard”, respectively. Toxic effects are accounted for by directly modifying energy fluxes, material fluxes, and/or the hazard rate ([7], [18], [19]).

In this paper, we develop a model to describe data on dissolved cadmium toxicity effects in *Pseudomonas aeruginosa* growth ([20]). These data are particularly interesting because they include those later phases of population growth that are required to estimate parameters pertinent to the hazard rates. Our model accounts for ROS production, identified by many authors as the primary cause of negative effects of various types of chemical toxicity ([21]), including cadmium toxicity ([2], [22]). The new work starts from a “standard” DEB model elucidated by Kooijman (2010 [16] - see ch. 6), and Sousa *et al.* (2008 [23]). Describing the population size trajectory in the absence of toxicants required explicit representation of environmental degradation associated with the bacterial metabolism. Modeling toxic effects involved considering acclimation to the toxic metal, effects of toxicant on assimilation, and toxicant-induced production of ROS leading to increase in mortality (section Model Description). We first present model simulations of the control and exposure treatments, and compare the maximum simulated growth rates to those calculated from the calibrated data using methods described in section Methods. Next, we test the ability of the model to predict the bacterial growth process for treatments not used in parameter estimation. Finally, we compare predicted aging acceleration to ROS measurements from Priester *et al.* (2009 [20]). Additional details of the model, additional results, and possible extensions of the model can be found in Information S1. We discuss the results and their implications, and suggest types of data that would greatly improve our ability to understand and model cellular processes.

### Model Description

**Figure 1:**
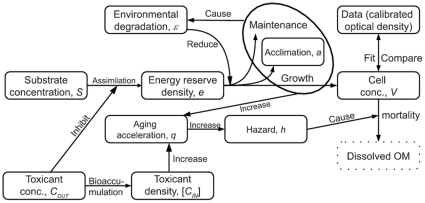
Outline of the model. Bacteria assimilate substrate into energy reserves, which are utilized to fuel growth (linked to increase in cell concentration), maintenance and acclimation. Products related to respiration degrade the environment, reducing the ability of bacteria to utilize energy reserves. Both toxicants and degradation of the supernatant inhibit assimilation of the substrate, and absorbed toxicants bioaccumulate in bacterial cells. Toxicants in the cell, as well as the cell's own metabolism, increase aging acceleration (by creating damage-inducing compounds), thus increasing the hazard rate, and mortality.

We started with the “standard” DEB model for bacterial production described by Hanegraaf and Muller (2001 [24]). Bacteria are assumed to be 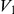-morphs, i.e. they can be approximated with a rod-like shape with area proportional to its volume. With this simplification, the same equations describe the dynamics of individual cell, and population growth without mortality [16]. Energy flows in our model are illustrated in Figure 1, the state variables and equations in Table 1, and parameters and their fitted values in Table 2. Further details on the equations are in Information S1.

**Table 1:** Summary of state variables, units, and dynamic equations.

State or helper variable	Units	Symbol	Dynamics
substrate concentration	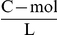	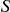	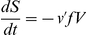
scaled energy density	n.d.	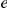	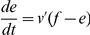
hazard rate	h 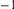	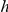	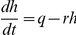
scaled functional response	n.d.		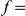 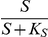
bacterial production rate	h 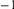		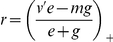
toxicant cellular density	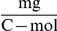	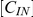	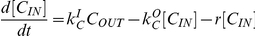
acclimation energy density	n.d.	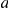	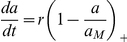
cell structure conc.	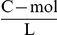	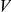	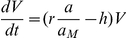
aging acceleration	h 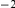	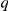	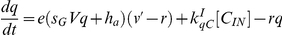
environmental degradation	n.d.	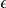	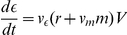
conductance modified by (6) and (8)	h 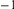		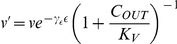

Bacterial production rate and scaled functional response (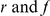) are not state variables, but have been defined separately for brevity. Non-dimensional variables have been labeled ‘n.d.’. Subscript ‘+’ signifies that only positive values of the expression are considered, with the expression set to zero if its value turns out to be negative.

**Table 2:** Parameters, units, and fitted values.

Parameter	Units		Std. value
Energy assimilation and utilization parameters			
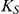	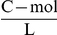	half-saturation constant	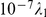
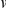	h 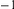	energy conductance	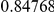
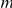	h 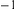	maintenance rate	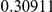
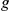	n.d.	investment ratio	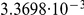
Parameters affecting increase and effects of aging acceleration			
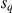	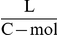	multiplicative stress coeff.	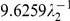
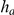	h 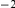	Weibull aging acceleration	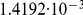
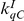	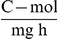	toxicity	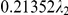
Toxicological parameters			
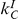	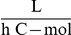	Cadmium influx coeff.	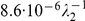
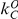	h 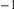	Cadmium efflux coeff.	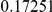
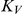	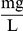	Noncompetitive inhibition coeff.	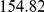
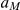	n.d.	Required acclimation energy	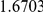
Other parameters			
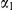 , 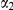	n.d.	calibration coefficients	
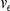	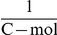	environmental degradation coeff.	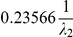
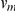	n.d.	maintenance respiratory coefficient	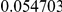
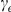	n.d.	environmental degradation effect coeff.	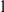

Concentration denotes an amount of per volume of substrate, density denotes an amount per structural volume of bacteria, and n.d. stands for ‘non-dimensional’. Coefficient 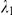 scales initial substrate C-mol concentration to unity, and 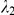 structural cell C-mol to calibrated optical density.

In the absence of a toxicant, a “standard” DEB model requires four state variables to describe a bacterial cell: structural volume, energy reserve density, density of damage inducing compounds, and hazard rate, a measure of mortality risk. Substrate concentration in the supernatant is the only state variable characterizing the environment. In the subsequent exposition, we distinguish between cellular and supernatant quantities by using the term “concentration” for mass and volume per volume of supernatant, and “density” for cellular quantities per unit of structural volume unless otherwise noted. Following standard practice in the DEB literature (e.g. [16]), we reserve the notation [..] for densities.

The standard model can easily be extended to include bioaccumulation of toxicants ([16], chapter 6). We further extend the standard model to include important features of bacterial physiology and the environment, especially relevant to toxic effects and later stages of growth. Extensions of the standard model considered here are: (i) additional terms in the equation for the hazard rate; (ii) a variable representing environmental degradation; (iii) mechanistic description of toxic effects linked to increase in ROS production and aging acceleration, and to non-competitive inhibition of transport channels; (iv) implementation of lag time using a concept of energy required for acclimation.

### Bioaccumulation

Our bioaccumulation model is similar to that used in previous work ([16], Section 6.3). Cadmium salts dissolve and release Cd(II) ions which may form other cadmium complexes, all assumed to exert a collective effect on cells. We use the total cadmium concentration, 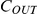, as the measure of environmental dissolved cadmium concentration, and the total cadmium density, 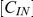, as the measure of bioaccumulated cadmium mass per unit of bacterial structural volume. For simplicity, and consistent with the experimental data for which the modeling herein is compared, 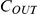 is assumed to be constant.

Diffusion or active transport, proportional to the surface (membrane) area of the bacteria and the outside concentration (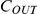) with a constant of proportionality 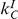, increases cadmium density (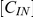). Furthermore, we assume that the toxicant is eliminated from the cell by virtue of efflux pumps, or complexed and thus becoming unavailable, with a rate constant 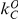. The rate of change of cellular Cd concentration is then
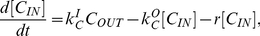
(1)where the last term on the right-hand side is dilution by growth proportional to the bacterial production rate, 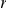 (Table 1; for details see Information S1, section Model of bacterial growth dynamics).

### Aging acceleration

DEB theory recognizes free radicals and reactive oxygen species (ROS) as the main cause of production of damage-inducing compounds (e.g. DNA damage) and, consequently, aging and cell damage ([16], pp. 209–214). Aging acceleration is a scaled measure of accumulated level of damage-inducing compounds. Just as the accumulated damage-inducing compounds determine the rate of increase of cellular damage, aging acceleration determines the rate of increase of the hazard rate. The acceleration can be interpreted as increasing due to two components: a multiplicative component corresponding to Gompertz, and an additive component corresponding to Weibull mortality (see [25] and section 6.1.1. in [16] for a detailed discussion on the differences). Both components are assumed to be proportional to the catabolic flux of the organism; the Gompertz term is also proportional to the already reached aging acceleration.

Dilution by growth is the only mechanism that reduces the average aging acceleration reached as the result of the metabolism and cadmium exposure (see Dilution by growth terms section of Information S1 for details on how the dilution is accounted for). Starting with equation (6.2) in Kooijman (2010 [16]), and modifying catabolic flux formulation to account for the bacterial shape, the rate of change of aging acceleration, 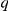, is
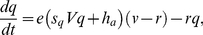
(2)where 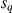 is a multiplicative stress coefficient, and 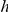 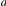 is the aging (Weibull) acceleration as defined in Kooijman (2010 [16], pp. 211). The stress coefficient is a constant accounting for the ability of the culture to dilute by growth in a given environment, and is proportional to the Gompertz stress coefficient (denoted 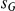 in Kooijman (2010 [16])).

We extend the formulation to include effects of exposure assuming that the toxicant produces ROS and, therefore, increases aging acceleration in proportion 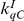 to bioaccumulated toxicant concentration (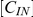). In principle, ROS could be only one of the ways by which toxicants affect aging acceleration, but the linear term describes other mechanisms as well. For example, cadmium increases aging acceleration by inhibiting DNA repair ([12], [15]), but the effect can be described by the same term. With the additional term, the rate of change of 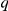 becomes
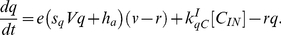
(3)


### Hazard

The hazard rate, 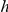, is the probability per unit time of dying at time 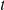:
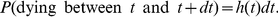
(4)According to DEB theory ([16]), the hazard rate changes in response to the aging acceleration:
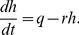
(5)


The second term on the right-hand side of (5) represents dilution by growth (for details, see Information S1, section Dilution by growth terms). Unlike multicellular organisms, which reduce the proportion of damaged cells in the whole organism as the organism grows, unicellular organisms cannot dilute hazard by growth (see [16], section 6.1.2). We, however, choose to include the dilution by growth term because we use the DEB model for a **population** of unicellular organisms which is, in its entirety, much more similar to a multicellular than an unicellular organism.

Toxic effects on mortality in previous DEB models are accounted for by adding to 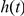 a term proportional to the difference between 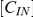 and a no-effect concentration ([26]). We, however, account for toxic effects in a different way. Experiments show that cell death rate correlates with ROS density, even when comparing different mechanisms of ROS production ([27]). This implies that *any* source of ROS contributes to increase in aging acceleration and, therefore, the hazard rate, and the mortality. We take the implication further by assuming that, rather than affecting cell death directly, *all* contributions of toxicants (e.g. dissolved cadmium) to cell death come from the ROS produced by the toxicants, and the resulting increase in aging acceleration. Therefore, any effect the toxicant may have on hazard is accounted for through increase in aging acceleration, and equation (5) captures the effect of toxicants on mortality.

### Environmental degradation

In addition to depleting nutrients, bacteria may degrade their environment in other ways. Given the large cell densities reached in bacterial cultures, the negative effects have the potential to be significant. Yeast, for example, degrade their environment by producing alcohol, and levels of dioxygen in post-exponential growth in plate cultures of aerobic bacteria have shown to be immeasurably small ([28]). In conditions of this experiment, dioxygen limitation is a more likely cause of environmental degradation than a toxic intermediate because CO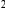 is the final product of aerobic mineralization of the provided organic carbon by *P. aeruginosa*. Modeling the exact mechanism of environmental degradation is, however, beyond the scope of this paper. We therefore account for effects of environmental degradation in a general way.

There are a number of ways environmental degradation can affect cellular processes. Regardless of the actual mechanism, we suggest that respiration is a good surrogate measure of environmental degradation. The conjecture is obvious for the two examples given above (alcohol production and dioxygen limitation), even though aeration through the air/supernatant interface has not been accounted for. In microplates, as evidenced by dioxygen concentrations presented in Kocincova *et al.* (2008 [28]), aeration seems not to be significant; in other experimental setups it might be and needs to be taken into the account if lack of oxygen is the cause of environmental degradation.

One of the simplest ways to model the decline in organism's ability to acquire and utilize energy due to environmental degradation is to exponentially reduce the parameter characterizing energy conductance (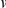):
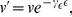
(6)where 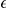 is a measure of environmental degradation (see below) and 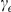 characterizes the strength of the metabolisms' response to environmental degradation. The exponential term in (6), 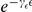, is a measure of environmental quality compared to the initial quality.

When a metabolite is responsible for the degradation, we suggest that respiration (which in DEB theory is a weighted sum of terms proportional to growth and maintenance rates - see Kooijman, (2010 [16], ch. 4.4)) is a better measure of metabolite production than the total energy utilization (catabolic flux) because the latter includes materials invested in growth which - because they are utilized elsewhere - cannot contribute to the production of metabolites or oxygen consumption. We therefore introduce a measure of environmental degradation, 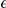, whose rate of increase is proportional to a weighted sum of growth and maintenance rates:
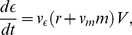
(7)where 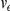 and 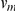 are environmental degradation and maintenance respiration coefficients. Since we do not measure environmental degradation, we can scale 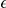 by 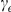 without a loss of generality. Hence, 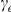 can be set to unity.

### Effect of dissolved cadmium on assimilation

Cadmium ions in the supernatant can reduce substrate uptake and, therefore, assimilation by affecting cation cross-membrane transport sites ([29], [30]). The magnitude of the influence depends on the concentration of cadmium ions in the supernatant. We assume the magnitude can be accounted for using standard non-competitive inhibition affecting assimilation and, therefore, energy conductance (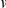) - see Muller *et al.* (2010 [19]). Reduction of conductance 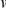 to 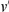 due to supernatant cadmium ion concentration 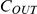 is then described by the formula:
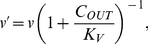
(8)where 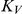 is the inhibition constant. Effects on 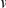 described by equations (6) and (8) are multiplicative (see Table 1). We considered the possibility of effects on other physiological rates, but did not include them in the model. See Information S1 for additional discussion.

### Acclimation to cadmium exposure

When exposed to a new environment, bacteria require some time to acclimate before starting to grow at a maximal rate; when retardation in growth is significant, it is referred to as the lag time. Some authors suggest that the lag time may be due to diversion of energy from bacterial growth to changes in structure and function. In case of cadmium exposure, this could entail cadmium efflux pumps and damage repair machinery; for example, Gibbons *et al.* (2011 [31]) demonstrate the effects of energetic cost of cadmium efflux machinery on bacterial yield.

Lag time has been previously modeled as a consequence of work needed for overcoming a hurdle ([32]), suppression of division until cells reach an environment-dependent size ([33]), or an empirically derived function of previous growth and environmental states ([34]).

DEB models are especially well suited to quantify acclimation dynamics because they quantify energy flux available to acclimation as a function of current environmental conditions, growth status, and energy availability (e.g. [35]). Once the available energy is known, the deficit of energy committed to growth and, consequently, retardation in growth can be estimated.

We start by assuming that, initially, the whole available energy flux (equal to the bacterial production rate, 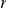) is diverted to acclimation. When partially acclimated cells divide, their daughter cells are assumed to be acclimated to the same degree. To ensure a smooth transition between acclimation and exponential growth phases, the diverted energy flux needs to decrease with the level of acclimation. We assume that this decrease is linear. Hence, if 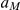 is the (non-dimensional) energy density required for acclimation, and 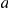 the corresponding cumulative energy density invested in acclimation at any given time, the rate of change of acclimation energy density is:
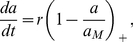
(9)where the subscript ‘+’ denotes that only positive values should be considered, and the expression evaluated as zero otherwise (see Information S1, section Acclimation, for derivation).

## Results

### Control

**Figure 2:**
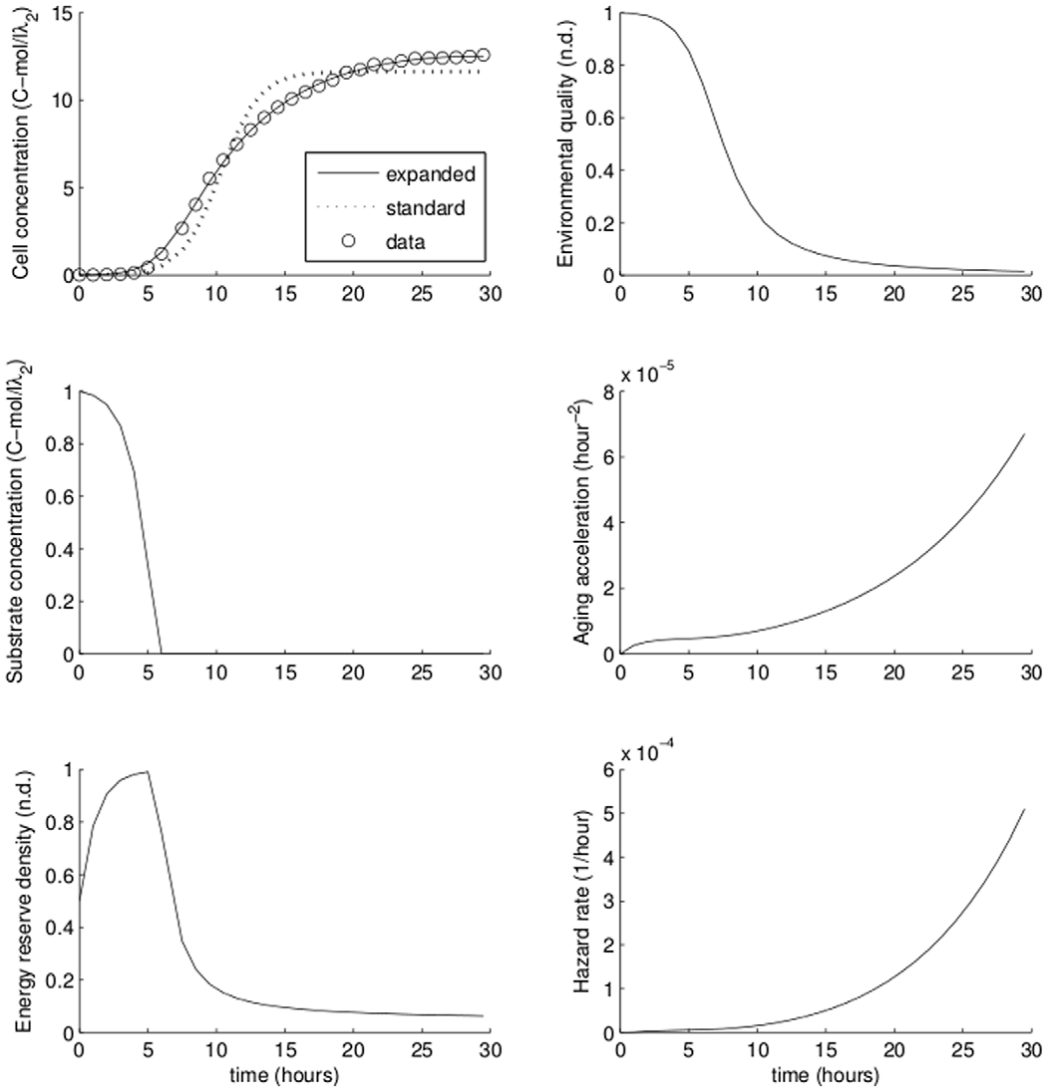
Simulating the control. Cell concentration and all state variables of the model except acclimation and bioaccumulation (not applicable for control). Upper left corner: data (circles), best fit of the standard model (dotted line) and best fit of the model extended by including environmental degradation (solid line). See text for discussion.

A model fit to control treatment (Figure 2) allows an overview of the model outputs. There are no surprises: the model fits the data extremely well (upper left plot, solid line). Scaled energy reserve density increases rapidly from the assumed initial value of 0.5 to 1 (bottom row, left panel), and then decreases because growth and maintenance utilize reserves as substrate disappears six hours into the experiment (middle row, left panel). Since exponential growth is possible only while energy reserves are constant, energy reserve density dynamics suggests true exponential growth is only occurring during hours 3–5 of the control treatment. Environmental degradation (top right panel) reduces environmental quality and, consequently, energy conductance only slightly during the first five hours, but picks up rapidly thereafter; at about 7.5 hours, the degradation is strong enough to reduce energy conductance by 50%. In line with expectations, aging acceleration increases rapidly after 10 hours when growth slows down (middle row, right panel), and so does the hazard rate (lower right panel).

Examination of the standard and expanded model fits (Figure 2, upper left panel, dotted line) using semi-logarithmic plots (not presented) shows that the fit of the standard DEB model underestimates the exponential growth rate, overestimates the rate at which cells stop growing (growth cessation), and underestimates the final bacterial cell concentration (bacterial yield). Including environmental degradation in the model enables a good fit to data primarily by slowing down the cessation of growth (Figure 2, upper left panel, solid line).

### Toxic response fit

**Figure 3:**
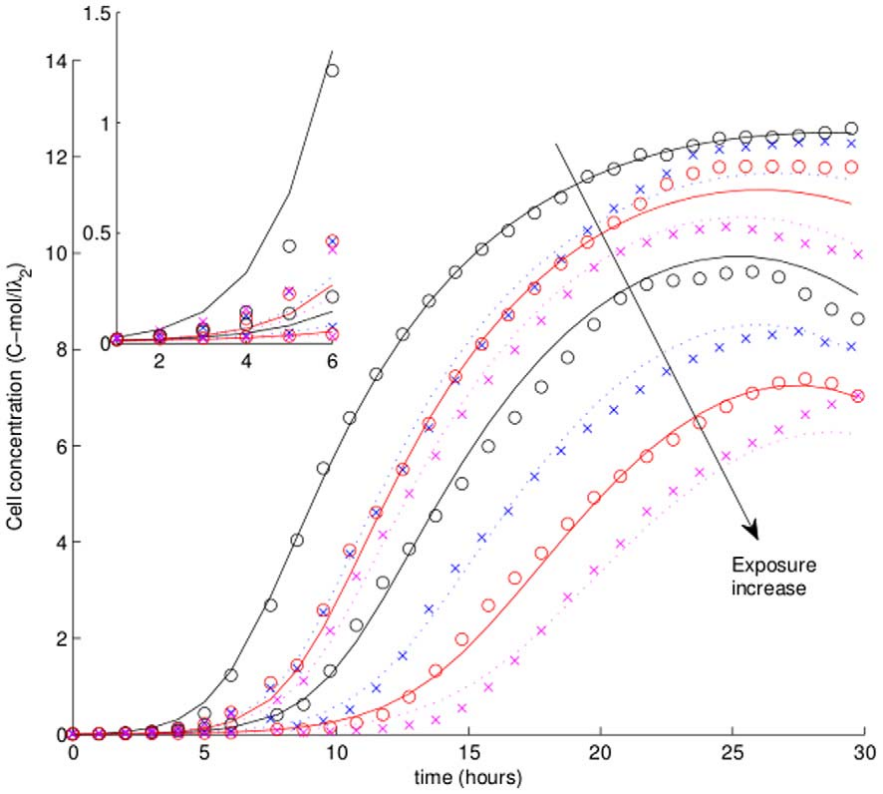
Simulations of all treatments with a single parameter set. Best fit set of parameter values used (listed in Table 2). The inset is showing the first 5 hours of the experiment.

**Figure 4:**
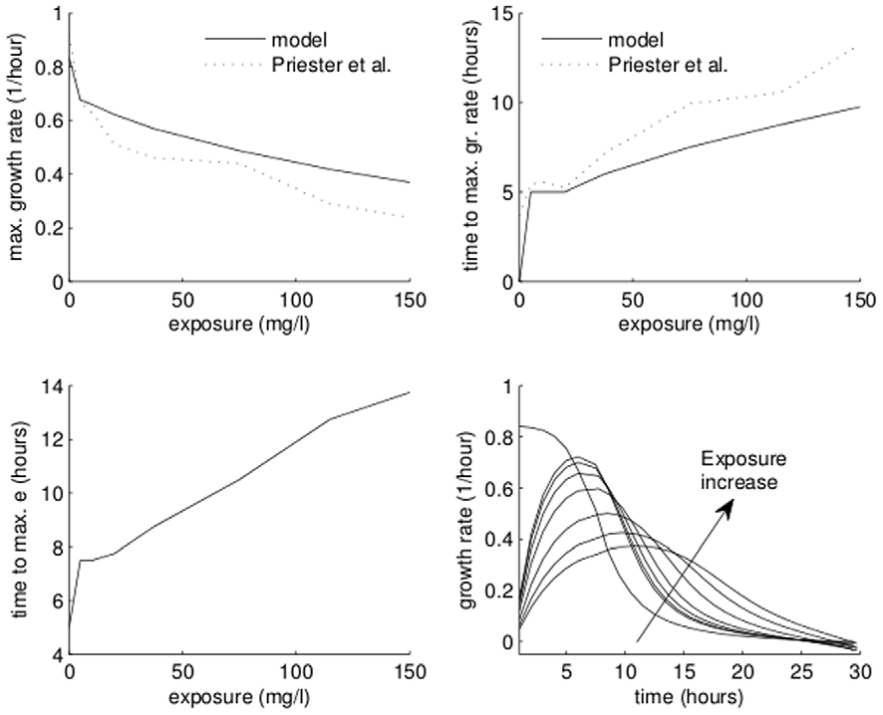
Overview of population dynamics. Top panels show dependence of maximum growth rate (left) and time to maximum growth rate (right) calculated from the model (solid line) and measured by Priester *et al.* (2009 [20]) (dotted line). Bottom left panel shows time to maximum energy density as a function of exposure concentration. Bottom right panel shows growth rates for all treatments during the first 30 hours of the experiment.

Concurrent fit of all treatments shows a satisfactory fit to the observations (Figure 3). The model underestimates yield for low toxicant concentrations (5 and 10 mg/L), but captures the most important features of higher concentration treatments. Even though the required acclimation energy density is the same for all treatments, time until near-exponential growth increases with exposure because it takes a longer time for bacteria to reach maximum energy density (Figure 4). The fit for pre-exponential phase (inset in Figure 3) is reasonable, but growth for lower exposures is slightly underestimated.

### Growth rate and energy density

Predictably, maximum exponential growth rate (Figure 4, upper left panel, solid line) decreases with exposure, roughly following the bi-phasic pattern observed by Priester *et al.* (2009 [20]) (same panel, dotted line). Exposure increases the time at which growth rate (upper right panel) and energy reserve density (lower left panel) reach the maximum. Time to maximum growth rate increases with exposure slightly slower than lag time (dotted line, upper right panel). The distribution of growth rates as a function of exposure (lower right panel) shows slower changes in growth rate with increased exposure.

### Aging acceleration dynamics

ROS levels in cultures can increase or decrease from the initial level (e.g. Figure 6 in [36]), depending on whether the ROS production rate is higher or lower than the dilution by growth. In some cases (e.g. Figure 6 in the control in [36]), the cells may be able to balance the ROS levels so that there is no net increase throughout the experiment. When the cells are not able to balance ROS levels due to environmental conditions (e.g. additional ROS production due to toxicants), the levels start to rapidly increase as the population growth rate decreases (e.g. phenazine-1-carboxylic acid treatments in Figure 6 in [36]). Additional growth cycles could again dilute ROS levels, but in a batch culture ROS are expected to follow patterns similar to those observed by Denning *et al.* (2003 [36]). Since the dynamics of aging acceleration in our model highly reflects ROS dynamics, it is also to be expected that the aging acceleration follows the same qualitative patterns as ROS.

**Figure 5:**
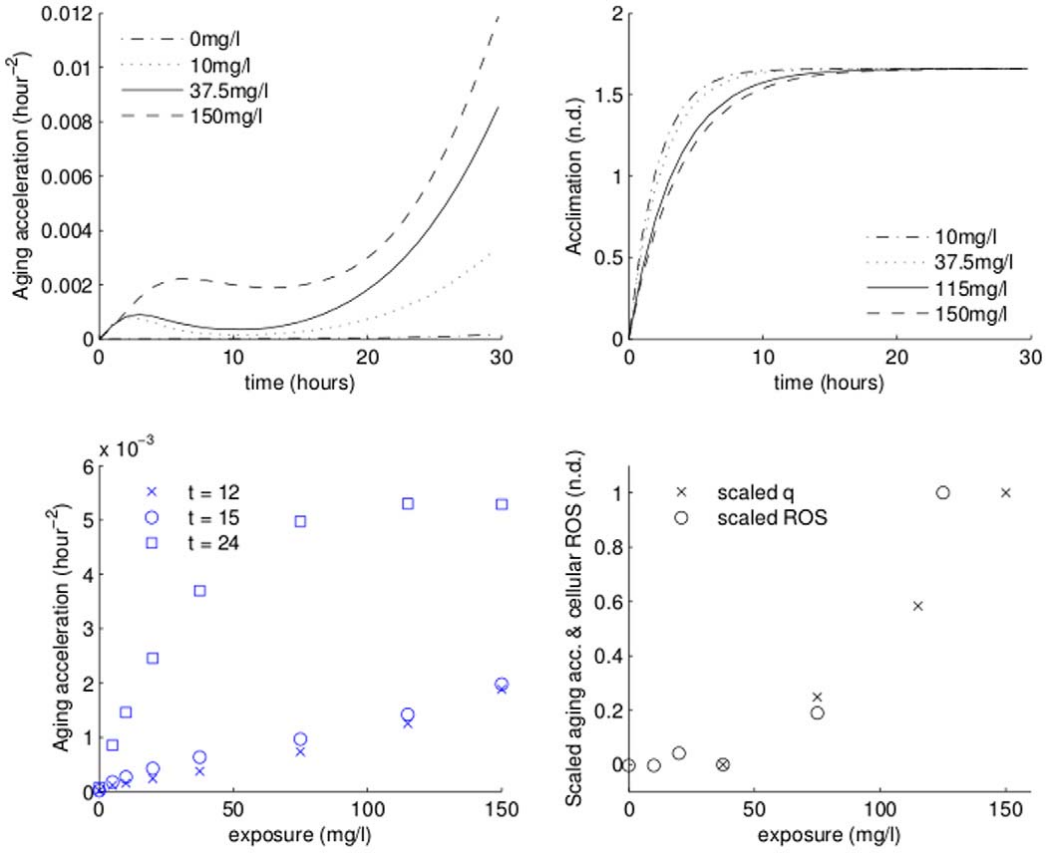
Dynamics of aging acceleration. Top: aging acceleration (left), and acclimation (right) for select (see legend) treatments. Bottom: aging acceleration for all treatments at 12, 15, and 24 hours (left), and comparison between scaled measured ROS and predicted aging acceleration (right).

Aging acceleration indeed follows the expected general pattern (Figure 5, top left panel). The control treatment retains a very low aging acceleration throughout the experiment, while those of cadmium treatments increase during the initial acclimation phase, decrease (due to dilution by growth) when the population starts growing, and increase again as dissolved cadmium bioaccumulates (and ROS production rapidly increases). Cells exposed to lower concentrations are able to commit more energy per unit time to acclimation and, therefore, acclimate faster (upper right panel, Figure 5). In general, aging acceleration increases with exposure concentration (lower left panel, Figure 5). The lower right panel (Figure 5) compares simulated aging acceleration and ROS measured at 15 hours by Priester *et al.* (2009 [20]). To facilitate comparison, in both cases we have assumed that the value for 40 mg Cd/L represents the detection limit, and scaled both sets of data by the maximum value.

### Predictive ability

One of the more important reasons for developing mechanistic models is to extrapolate to behaviors in conditions beyond the range used for parameter estimation. In this section, we test the ability of our model to predict the growth of bacteria exposed to levels of cadmium not used in parameter estimation.

**Figure 6:**
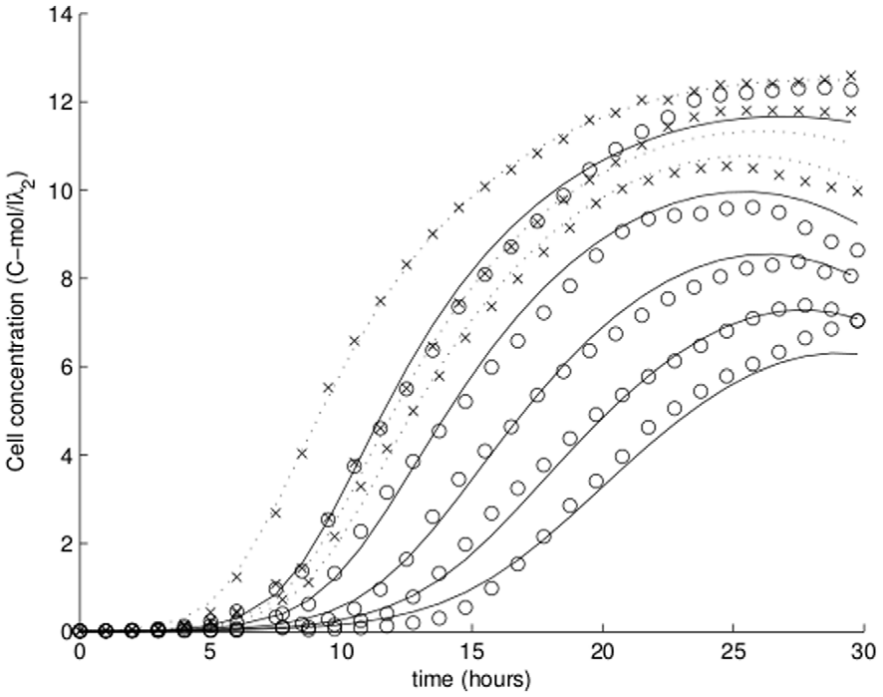
Predicting high exposures. Exposures of 37.5, 75, 115, and 150 mg/L predicted using fits only of data on control and low exposures (10 and 20 mg/L). Data points marked with ‘x’: data used in fitting; ‘o’: data used for comparison only. Dashed line: fitted treatments (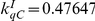, 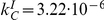, 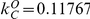, and 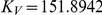). Solid line: predicted treatments.

We start by fitting the model using just the control and two low-concentration treatments, then assess how well the model predicts other treatments. One caveat is that the fitting procedure requires initial guesses of parameter values; if the guesses are too far off the mark, the fitting procedure cannot converge. Using the converged parameter values as initial guesses, however, defeats the purpose of the exercise. To ameliorate this problem, we changed free toxicology parameters (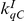, 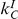, 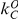 and 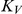) by a random percentage as large as 50% from the values used in Figure 6. Next, we fitted the parameters to a subset of treatments using the changed values as initial guesses. We chose to keep the required acclimation energy constant because having to estimate five toxicological parameters would require a larger number of curves (but see Information S1).

Figure 6 contains an example where we fitted the parameters to 10 mg/L and 20 mg/L treatments. The prediction is satisfactory given that the highest predicted treatment is more than seven times higher than the highest treatment used to estimate parameters. Additional predictions are available in Figure S3 and Information S1.

## Discussion

The work reported in this paper extends the DEB modeling framework to include a mechanistic description of lag time, and account for toxic effects through the effects of toxicants on aging acceleration (scaled damage-inducing compound density). It also includes a novel connection between the aging acceleration and physiological rates that feature in DEB models. Our primary aim was to find a minimal mechanistic representation consistent with the results of one set of experiments ([20]), and to determine some of the model's predictive capabilities. We now discuss the key features and limitations of our model, note some practical points concerning the design of future experiments, and consider the wider implications of our work. While reading the discussion, the reader should keep in mind that damage-inducing compounds as defined in the DEB theory are the basic concept, while aging acceleration is an interpretation which connects the DEB formalism to population-level processes; the two concepts only differ in scaling and are, therefore, mathematically equivalent.

Development of the model reported here was preceeded by work on a much more complex model that, inter alia, distinguished “active” and “inactive” cells, and implemented toxic effects on additional physiological processes such as maintenance, costs of growth, and cell lysis. We first created and fitted a model based on one set of assumptions, then repeated the process with a slightly different set of assumptions until we reached a balance of model complexity and ability to capture main features of the data. The final model reached in this iterative process has a fair number of parameters, with which, if the model were wholly empirical, it might have been possible to fit practically any set of curves. However, the mechanistic nature of the model, and the need to satisfy eight curves at the same time with a single parameter set, severely limits the shape of the growth curves that can be obtained (see discussion in section Methods, and possible extensions of the model and alternative model assumptions in sections “Differentiating between active and inactive cells”, and “Effects of damage-inducing compounds” of Information S1). This limitation enables exploration of alternative hypotheses: if a particular growth curve cannot be obtained by the model, at least one of the assumptions must be wrong.

Modeling environmental degradation emerged as a key component of the model as this assumption was required to describe the control treatment. A DEB model (Figure 2, upper left panel, dotted line) that lacked this component could not explain the slow cessation of growth observed in the control treatment, while a model with environmental degradation could. Our treatment of environmental degradation was of necessity phenomenological, but we note that the pattern of inhibition by environmental degradation (Figure 2, top right panel) closely matches dioxygen concentration in microplate wells measured by Kocincova *et al.* (2008 [28], Figure 8). Alternatives do, however, exist; for example, we were also able to capture the slower cessation of growth using an assumption that substrate is of heterogeneous quality, and that the half-saturation constant (handling time) increases for substrates of lower quality. Final substrate levels resulting from these assumptions were, however, implausibly high (more than 95% of the substrate could not be utilized). Given the realistic environmental degradation profile, and our reluctance to assert that bacteria cannot utilize more than 95% of the substrate, we adopted environmental degradation as the key mechanism of slower-than-expected growth cessation. Direct information regarding changes in the environment would likely enhance the diagnostic and predictive power of future models.

We discovered that the toxic effects on bacterial assimilation and hazard rates were alone sufficient to explain effects of exposure on population dynamics of *P. aeruginosa* observed by Priester *et al.* (2009 [20]) (Figure 3). The sole direct effect of dissolved cadmium was to affect aging acceleration (i.e. damage-inducing compounds) through production of ROS, which mediated all intracellular toxic effects (equations (1) and (3)). Of course, damage-inducing compounds resulting from exposure could also affect other energetic processes in the cell such as increasing maintenance and decreasing growth efficiency (see [2] and the Introduction), but it was not essential to consider these mechanisms to fit the available data. We recognize that the energetic implications of damage-inducing compounds are ultimately related to biomolecular processes such as protein turnover, efflux protein assembly, and cross-membrane gradient maintenance. Information on these molecular processes could - and should - influence the choice of processes to be included in future models.

There are some practical messages from the modeling that are relevant to future experimental studies that will be interpreted using DEB models. One obvious example is the importance of obtaining some measurements that allow calibration of data on proxies (such as optical density) for the DEB variables. Less obvious is the need for careful experimental design in studies intended for comparison of intracellular quantities such as ROS levels, for example the data shown in Figure 5. It is a common practice to take samples for ROS measurements at the same time in the experiment (e.g. 15 hours in Priester *et al.* (2009 [20])), and then draw conclusions on effects of exposure on ROS production. Simulations suggest that such a procedure may carry a number of pitfalls. Specifically, because of dilution by growth effects, ROS density depends on growth phase as well as the exposure and the bioaccumulated toxicant (Figure 5). If the population grows faster than cells accumulate ROS, ROS (and, possibly, damage-inducing compound) density decreases. Consequently, comparing a lower concentration treatment which entered a stationary phase with a higher concentration treatment, which had a longer lag phase and is growing exponentially, may give a false impression that the higher concentration treatment suffers less impact of exposure. Comparing ROS measurements in stationary phase carries similar pitfalls: accumulated ROS depends on the duration of the stationary phase and the rate of ROS creation. Similar considerations should be taken into account when considering bioaccumulation (Figure S2 and Information S1), where dilution by growth effects reduce total cadmium in cells. The observed bioaccumulation there suggests that most of the differences during mid-exponential growth phase (hours 4–13) come from bioaccumulation during the acclimation phase.

Investigating ROS dynamics during the acclimation phase could provide insights into reasons behind the observed lag phase. Our assumption that all treatments require the same acclimation energy because they need to produce the same molecular machinery may be wrong if cells can adjust the magnitude of the response. For example, the lowest two concentration treatments give maximum cell densities significantly lower than the observed, partly because energy spent on acclimation reduced total energy available for growth. If lower exposure concentrations required less acclimation, the maximum cell density would have been higher. This would also reduce the underestimates of pre-exponential growth for low exposures.

Our framework can be expanded to link actual molecular mechanisms of ROS creation and neutralization to the production of damage-inducing compounds (and increases in aging acceleration). Currently, we followed DEB theory in assuming a fairly simplistic dynamics of ROS: each ROS produced gives a fixed contribution to aging acceleration. We could instead differentiate between reactive species and their effect on aging acceleration to consider how different environmental conditions affect bacterial growth: certain reactive species could produce more damage-inducing compounds than others, while some reactive species could cascade through multiple forms before being neutralized, increasing aging acceleration with each transformation. Different toxicants could produce different numbers and species of ROS. The physical location of the ROS could also be taken into the account: damage to the membrane affects different processes than damage to the DNA. Available data and knowledge, however, simply do not allow such elaborations.

Clearly, additional data are necessary to tune the current model into a reliable theoretical framework which would enable accurate predictions. Ideally, we would want data sets that allow for independent testing of model assumptions and reduce ambiguity in parameter estimation. Data that would have greatly helped in the present work include: media characterization throughout the experiment to determine possible mechanisms of environmental degradation, including a time series of dioxygen availability, supernatant ROS, and pH; time-series of cellular ROS and/or other reactive species for all or most treatments to help determine initial delay mechanisms, validate assumptions on accumulation of damage-inducing compounds, and help estimate parameters; time-series of cellular and supernatant dissolved cadmium concentrations to fine-tune the dynamics of bioaccumulation; alternative methods of characterizing population size; any and all physiological processes, such as respiration, that can be measured.

Despite the shortcomings, our model represents a step towards understanding the connections between cellular-level processes and population-level consequences, addressing many of the limitations of traditional approaches to ecotoxicology identified by Baas *et al.* (2010 [37]). We were able to make testable predictions on bioaccumulation and time-dependent density of damage-inducing compounds, thereby enabling validation and hypothesis testing. This is important because toxic effects on bacteria are investigated almost exclusively utilizing population-level data. Furthermore, the model provided reasonably accurate predictions for exposures to almost an order of magnitude higher than those used in parameter estimation, demonstrating the potential of this approach.

## Materials and Methods

Details of the experimental setup are in Priester *et al.* (2009 [20]) and are only briefly summarized in Information S1. Here we describe the theory and experimental setup we used to relate observables to state variables, and methods used to simulate and fit the model.

### Relating observables to state variables

Relating state variables to data is a crucial step in any modeling effort. State variables of substrate, energy reserves, and structural volume are in C-mol concentration or density; data are not.

Substrate concentration is unknown, but we can assume all treatments have the same initial substrate concentration. Therefore, we can use a scaling factor, 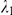, to scale C-mol of substrate per unit of volume of supernatant to 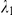
C-mol per unit of structural volume. Since 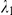 is unknown, we can without a loss of generality pick such 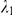 that 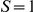 at the start of the experiment.

Relating data to structural cell volume is a two-step process. We need to relate the optical density (OD) measurements non-linearly related to cell concentration to a measure of cell concentration, and then convert that measure into C-moles.

OD is often assumed to be proportional to cell density of active bacterial cells (also assumed by Priester *et al.* (2009 [20])), but this assumption only holds for a limited range of ODs. When OD is higher than approximately 0.8–1, cell density increases much faster than linearly with OD (see [38] for more details). Since data from Priester *et al.* (2009 [20]) included optical densities well into the nonlinear range (higher than 1.6), we calibrated the data. We used data from a dilution experiment (see below) to fit the parameters 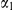 and 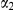 of empirical relationship between calibrated optical density, 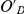, and measured optical density, 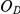:
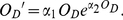
(10)The calibrated optical density (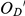) should be proportional to cell concentration and any other measure thereof (C-mol concentration, DNA concentration etc.). We do not have data on the relationship between calibrated OD and C-mol concentration, so we account for it with an unknown scaling factor, 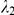 (structural volume in C-mol/L = 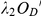).

We conducted a dilution experiment to calibrate optical density. Triplicate 7.5 mL cultures of *P. aeruginosa* in Luria-Bertani broth were inoculated from frozen stock (see Information S1 for more detail) and incubated at 30
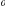
C/200 rpm in the dark for 24 h. The OD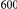 of each culture, as well as for diluted (1.67×, 3.33×, 4×, 8×, 10×, 80×, 100×, 800×, 1000×, 8000×, 10000×, and 80000×) samples, was measured. Sub-samples (10 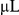) of the triplicate, undiluted cultures were stained with SYBR gold (Invitrogen, Carlsbad, CA) and cell counts were determined by epifluorescence microscopy. The total cell concentrations in undiluted 24 h cultures, along with the measured OD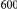 values for diluted samples, were combined to create a calibration for optical density (Figure S1 and Information S1). We ignored potential dependence of cell size on growth phase and any bias it may introduce.

### Fitting

The model presented in this paper has 13 free parameters and eight state variables, with only one state variable (or a combination of two if active and inactive cells are distinguished, see Information S1) directly corresponding to the measured data. We obtained the fitted parameter values in a two-step process. First, we fitted the DEB parameters that do not depend on toxicant levels (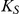, 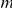, 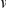 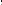 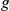, 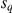, 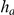, 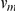 and 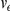) using the control data alone (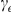 has been scaled to 1 and is not a free parameter). Then we fitted toxicant-related parameters (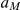, 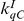, 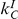, 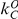 and 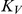) using data from all cadmium treatments. The fitting itself was done using MATLAB's nlinfit.m fitting routine (least-squares data fitting by the Gauss-Newton method). We simulated the model using proprietary code written in MATLAB, and calculated the square of the difference between observed and simulated cell concentration for a given set of parameters. We then adjusted the parameter values using Levenberg-Marquard step adjustments, and repeated the process until reaching convergence. When investigating predictive ability, we only used a subset of non-zero exposure treatments for fitting. In principle, parameters may have been estimated independently; for example, constants 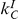 and 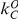 could have been estimated using methods such as those outlined by Hajdu *et al.* (2010 [39]). Such approaches could increase the usefulness of the theoretical framework outlined in this manuscript, but - due to their complexity - require separate efforts.

The mechanistic nature of the model and the need to satisfy eight curves at the same time with a single parameter set greatly constrained viable parameter values. We tried removing assumptions one at a time, and kept only those that had a significant impact on the goodness of fit (estimated subjectively); we were unable to obtain a reasonable fit after removing any one of the remaining assumptions. This, combined with a maintenance rate coefficient realistic for bacteria, gave us some confidence in the model; the fact that our predictions of aging acceleration reflected those expected from the observed ROS dynamics, and the ROS density measured by Priester *et al.* (2009 [20]) (see Figure 5 in Results, and section Relating observables to state variables) further improved our confidence.

Even though the least-squares method converged, there were a number of colinearities among parameter estimates. Furthermore, we cannot be certain that our routines did not locate a local minimum of the residual sum of squares. We investigated this by exploring the parameter space manually, but there remains the possibility that a better data fit than presented here exists. Finding it is, in our opinion, beyond the scope of the manuscript which showed that the model *can* explain the observations; obtaining data types identified as important by our research (see Discussion) should have much higher priority than further statistical refinement of the current analysis.

To minimize the possible influence that Cd salts had on optical density for low cell concentrations, we used the initial value of the structural volume of the control for all treatments. This decision recognized that all inocula came from the same stock. We arbitrarily assumed that cells had 50% of the maximum energy density when transferred from the inoculum by setting the initial energy density to 0.5. The precise value chosen did not affect our ability to fit the data. The initial substrate concentration was set to unity (without loss of generality - see above), and all other initial values (environmental degradation, bioaccumulated toxicant, aging acceleration, acclimation, and hazard) were set to zero.

## Supporting Information

Figure S1 **Comparison of raw (left panel) and calibrated data (right panel).**
(TIF)

Figure S2 **Bioaccumulation for 5 (lowest solid line), 10, 20, 37.5, 75, 115, and 150 (highest solid line) mg/L total cadmium.**
(TIF)

Figure S3 **Predictions using alternative data sets for fitting.** Data points marked with ‘x’: used in fitting; data marked ‘o’: not used in fitting. Solid line: simulations of data not used in fitting; dotted line: simulations of data used in fitting. Top left panel: predicting 5, 10, 20, 37.5 and 75 mg/L using 0, 115, and 150 mg/L. Top right panel: predicting 5, 10, 37.5, 115 and 150 mg/L using 0, 20 and 75 mg/L. Lower left panel: predicting 10, 20, 75, 115 and 150 mg/L using 0, 5, and 37.5 mg/L. Lower right panel: predicting 5, 20, 75, 115 and 150 mg/L using 0, 10 and 37.5 mg/L.
(TIF)

Information S1 **Supporting Information.** Additional results, overview of the basic DEB model and scaling, dicussion on how mortality affects the model equations, derivation of acclimation dynamics, and possible extensions of the model.
(PDF)
