# Supplementary figures and images for: Modeling Physiological Processes That Relate Toxicant Exposure and Bacterial Population Dynamics
Source: PLoS One. 2012 Feb 6;7(2):e26955. doi: 10.1371/journal.pone.0026955 (PMC3273461; doi:10.1371/journal.pone.0026955)

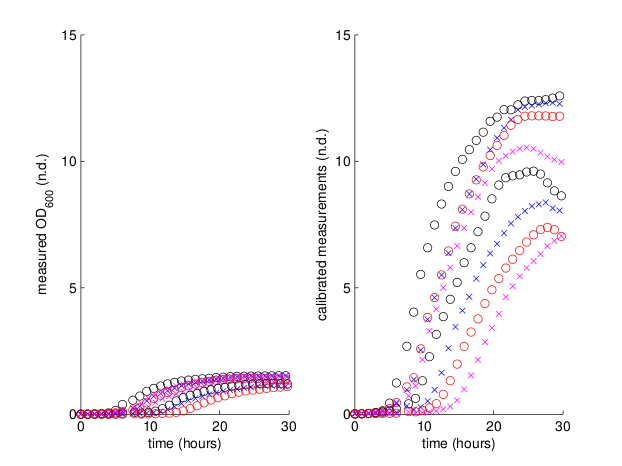

Supplement: Figure S1 — Comparison of raw (left panel) and calibrated data (right panel). (TIF) [file pone.0026955.s001.tif]

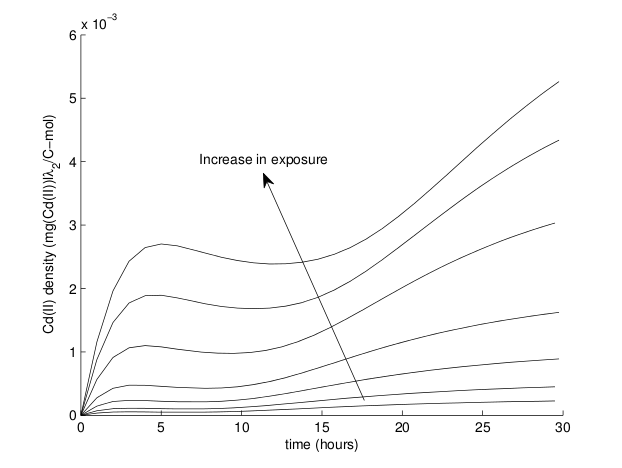

Supplement: Figure S2 — Bioaccumulation for 5 (lowest solid line), 10, 20, 37.5, 75, 115, and 150 (highest solid line) mg/L total cadmium. (TIF) [file pone.0026955.s002.tif]

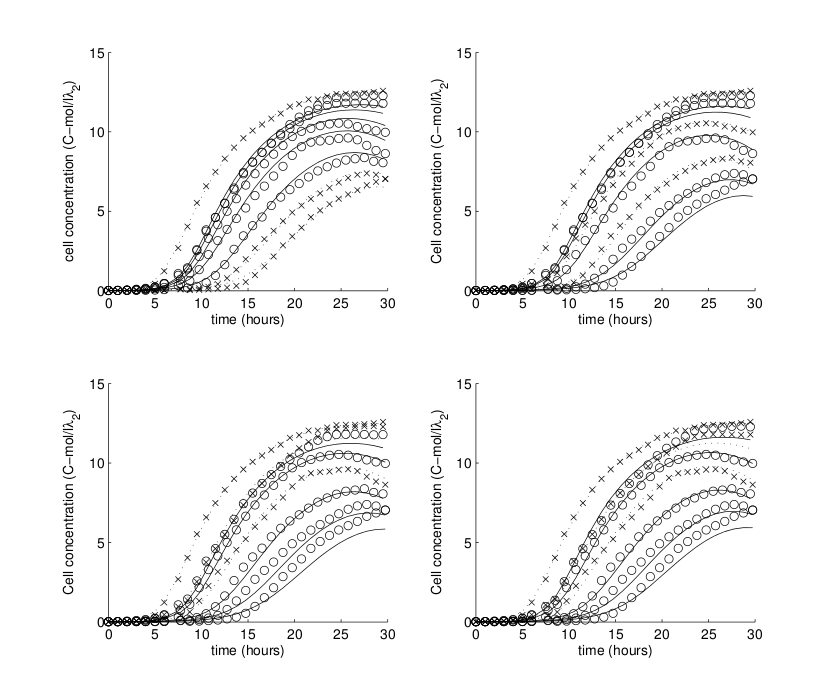

Supplement: Figure S3 — Predictions using alternative data sets for fitting. Data points marked with ‘x’: used in fitting; data marked ‘o’: not used in fitting. Solid line: simulations of data not used in fitting; dotted line: simulations of data used in fitting. Top left panel: predicting 5, 10, 20, 37.5 and 75 mg/L using 0, 115, and 150 mg/L. Top right panel: predicting 5, 10, 37.5, 115 and 150 mg/L using 0, 20 and 75 mg/L. Lower left panel: predicting 10, 20, 75, 115 and 150 mg/L using 0, 5, and 37.5 mg/L. Lower right panel: predicting 5, 20, 75, 115 and 150 mg/L using 0, 10 and 37.5 mg/L. (TIF) [file pone.0026955.s003.tif]
