# Supplementary material for: Modeling Physiological Processes That Relate Toxicant Exposure and Bacterial Population Dynamics
Source: PLoS One. 2012 Feb 6;7(2):e26955. doi: 10.1371/journal.pone.0026955 (PMC3273461; doi:10.1371/journal.pone.0026955)
Supplement: Information S1 — Supporting Information. Additional results, overview of the basic DEB model and scaling, dicussion on how mortality affects the model equations, derivation of acclimation dynamics, and possible extensions of the model. (PDF) [file pone.0026955.s004.pdf]

Supporting Information

Tin Klanjscek[1,2,*], Roger M. Nisbet [1], John H. Priester [3] Patricia A. Holden [3]
1 Department of Ecology, Evolution and Marine Biology, University of California Santa Barbara, Santa Barbara, California, 93106-9610, USA
2 Rudjer Boskovic Institute, Bijenicka 54, PO Box 180, Zagreb, Croatia, HR-10002
3 Bren School of Environmental Science and Management, University of California Santa Barbara, Santa Barbara, California, 93106-5131, USA
∗ E-mail: tin@irb.hr

The Supporting Information has six sections. First, we present results not included in the manuscript: effects of calibration (Figure S1), bioaccumulation patterns (Figure S2) and additional demonstration of prediction ability of the model (Figure S3). Next, in section Modeling bacterial growth dynamics, we describe the standard DEB model of bacterial production in terms of physical quantities, present a short description of scaling used to obtain the model equations presented in the manuscript, and discuss how mortality affects the model equations. Details of the derivation of equation 9 are presented in the section Acclimation. The next two sections present two major possible extensions of the model: differentiating between active and inactive cells, and effects of aging acceleration (or damage-inducing compounds) on parameters of the model not considered in the manuscript. Finally, we summarize the experimental setup from Priester *et al.* [1].

Additional results

For high optical densities, a nonlinear response of optical density to cell concentration can have a significant impact on perceived growth:actual cell concentration can be almost an order of magnitude greater than inferred from linear optical density (Figure S1).

The bioaccumulation pattern in Figure S2 shows that cadmium increases rapidly during the acclimation phase, then remains fairly constant or reduces slightly due to dilution by growth, and then increases as cells enter the stationary phase of growth. This would suggest that the bulk of the difference in bioaccumulation between treatments comes from the burden accumulated during the pre-exponential growth phase.

Figure S3 demonstrates the ability of the model to predict impacts of exposure. We have utilized the same methods as in the "Predictive ability" section of the results, but with alternative choices of treatments used for fitting to those used in Figure 6. All explored choices yield satisfactory predictive ability.

Model of bacterial growth dynamics

Figure 1 shows the model overview. To clarify units, and help link state variables used in the manuscript to physical quantities, we include a short summary of the standard DEB and scaling used in the manuscript. Kooijman [2] provides an in-depth overview of the DEB theory. Nisbet *et al.* [3] offer an ecologically oriented introduction to the theory and Sousa *et al.* [4] contains a compact, rigorous guide written in a much more mathematical style. More details on the standard approach to toxic effects can be found in Muller *et al.* [5] and Jager *et al.* [6].

Our model of bacterial population dynamics describes growth of a population by an individual DEB model using special morphological properties of *P. aeruginosa.* Their relatively small range of body sizes and rod-like shape enables us to approximate their growth by that of organisms whose surface area is proportional to their structural volume, called V1-morphs by Kooijman [2]. This means that - mathematically - we do not have to distinguish between cellular growth and cell division (see discussion on V1-morphs in Kooijman [2] (Section 4.2) for more detail).

We develop the model of bacterial population dynamics in two steps: first we develop a bacterial production model for cellular growth based on DEB theory and Hanegraaf and Muller [7], and then account for mortality to arrive at the population dynamics. The bacterial production model accounts only for the production term of growth, considering the whole population as one growing organism. Mortality cannot, however, be accounted for by the model of an individual because only a proportion of a population dies in a given moment, while an individuals dies only as a whole. When accounting for mortality (and associated material fluxes), we have to be very careful to understand which processes are affected.

Standard DEB model of bacterial production

Substrate uptake rate $\left(\frac{\textbf{C-mol}}{\textbf{volume time}}\right)$

$$I = [I_m] f V, \tag{S1}$$

$$f \equiv \frac{S}{K_S + S}, \tag{S2}$$

where $[I_m]$ is the maximum V-specific uptake rate $\left(\frac{1}{\text{time}}\right)$.

Energy assimilation rate $\left(\frac{\textbf{C-mol}}{\textbf{volume time}}\right)$

$$A = \kappa_V I \tag{S3}$$

$$= \frac{[A_m]}{[I_m]} I \tag{S4}$$

$$= [A_m] f V, \tag{S5}$$

where $[A_m]$, the maximum V-specific assimilation rate (1/time), is defined as the product of $[I_m]$ and an assimilation efficiency κ_V.

Rate of change of substrate $\left(\frac{\textbf{C-mol}}{\textbf{volume time}}\right)$ We could assume that food decreases due to uptake (I), or assimilation (A). This distinction is only important when we want to keep track of nutrients in physically meaningful units. Since our units of substrate are arbitrary (see discussion in section Relating observables to state variables), for simplicity we assume that food decreases due to assimilation:

$$\frac{dS}{dt} = -A. \tag{S6}$$

Rate of change of energy density $\left(\frac{\textbf{1}}{\textbf{time}}\right)$

$$\frac{d[E]}{dt} = [A_m] f - \frac{[A_m]}{[E_m]}[E], \tag{S7}$$

where $[E_m]$ is the maximum energy density, non-dimensional because both E and V are measured in C-mol per volume.

Bacterial production rate $\left(\frac{\textbf{C-mol}}{\textbf{volume time}}\right)$ Bacteria do not use energy for maintaining their temperature, so maintenance is just $[M]V$, where $[M]$ is the maintenance rate coefficient $\left(\frac{1}{\text{time}}\right)$. From the DEB theory, the catabolic flux, p_C, is

$$p_C = A - V\frac{d[E]}{dt} - [E]\left(\frac{dV}{dt}\right)_I, \text{ and (for } \kappa\text{=1)} \tag{S8}$$

$$p_C = [E_G]\left(\frac{dV}{dt}\right)_I + [M]V, \tag{S9}$$

where $\left(\frac{dV}{dt}\right)_I$ denotes the bacterial production term (and/or population growth without mortality). Rearranging gives

$$\left(\frac{dV}{dt}\right)_I = rV, \tag{S10}$$

where the bacterial production rate, r, is

$$r = \frac{[E][A_m]/[E_m] - [M]}{[E] + [E_G]}. \tag{S11}$$

Rescaling

Using standard compound parameters and scaling:

$$e \equiv \frac{[E]}{[E_m]}(\text{n.d.}) \quad v \equiv \frac{[A_m]}{[E_m]}(\text{t}^{-1}) \quad m \equiv \frac{[M]}{[E_G]}(\text{t}^{-1}) \quad g \equiv \frac{[E_G]}{[E_m]}(\text{n.d.}) \tag{S12}$$

model equations (S7) and (S11) turn into

$$\frac{de}{dt} = v\,(f - e)\,, \text{ and} \tag{S13}$$

$$r = \frac{ve - mg}{e + g}. \tag{S14}$$

Accounting for mortality

Hazard in DEB theory describes the probability of an individual dying. Although we model the population as an individual, we can't model mortality the same way because in a population, only a fraction of individuals die. We still consider the hazard as the probability of an individual dying, but interpret it as the mortality rate. The growth rate of the population is then the difference between the production (rV) and the mortality (hV) terms:

$$\frac{dV}{dt} = (r - h)\,V. \tag{S15}$$

Mortality affects dynamics of aging acceleration, hazard, and bioaccumulated toxicant.

Dilution by growth terms

When dealing with densities in the population-level model, we need to take care of dilution by growth. For a variable X (such as bioaccumulated toxicant, aging acceleration, and hazard) whose dynamics is described by

$$\frac{dX}{dt} = b_1 + b_2X, \tag{S16}$$

dynamics measured 'per structural volume' ([X]=X/V) is:

$$\frac{d[X]}{dt} = \frac{dX/dt}{V} + X\frac{dV^{-1}}{dt} \quad (S17)$$

$$= \frac{(b_1 + b_2 X)}{V} - \frac{X}{V}\frac{1}{V}\frac{dV}{dt} \quad (S18)$$

$$= b_1/V + b_2[X] - (r-h)[X]. \quad (S19)$$

If X describes toxicant bioaccumulation, acclimation, aging acceleration, or hazard, b_2 must include mortality ($-h$) because dead cells take the bioaccumulated toxicant, acclimation, aging acceleration and hazard with them. For example, bioaccumulation dynamics has:

$$b_1 = k_C^I C_{OUT} V, \quad (S20)$$

$$b_2 = -k_C^O - h. \quad (S21)$$

Inserting (S20) and (S21) into (S19) gives (1). For aging acceleration (q), $b_1 = \left(eh_a\,(v-r) + k_{qC}^I[C_{IN}]\right)V$, and $b_2 = es_q\,(v-r)\,V - h$, while for hazard (h), $b_1 = qV$, and $b_2 = -h$. Acclimation is derived in the section below.

Acclimation

From (S9), the energy available for somatic growth, J_G, is

$$J_G = [E_G]Vr. \quad (S22)$$

If a fraction κ_C of that energy is funneled to acclimation, the rate of change of acclimation energy committed, E_A, is the sum of newly mobilized energy committed to acclimation ($\kappa_C J_G$), and increase in E_A due to division of already partially acclimated cells which survived to divide (proportional to per-cell acclimation (E_A/V), and the population growth rate):

$$\frac{dE_A}{dt} = \kappa_C J_G + \frac{E_A}{V}\frac{dV}{dt}. \quad (S23)$$

In terms of acclimation energy density, $[E_A] = E_A/V$,

$$\frac{d[E_A]}{dt} = \frac{1}{V}\frac{dE_A}{dt} - [E_A]\frac{1}{V}\frac{dV}{dt} \quad (S24)$$

$$= \frac{1}{V}\left(\kappa_C J_G + [E_A]\frac{dV}{dt}\right) - [E_A]\frac{1}{V}\frac{dV}{dt} \quad (S25)$$

$$= \kappa_C \frac{J_G}{V} \quad (S26)$$

$$= \kappa_C [E_G] r. \quad (S27)$$

We choose the simplest form for κ_C that maximizes the flow of energy to acclimation when $[E_A] = 0$, and causes the flow to cease when acclimation energy has reached its required value, $[E_A^M]$:

$$\kappa_C = \left(1 - \frac{[E_A]}{[E_A^M]}\right) \quad (S28)$$

$$= \left(1 - \frac{a}{a_M}\right), \quad (S29)$$

where $a = [E_A]/[E_G]$ is the scaled acclimation energy density, and $a_M = [E_A^M]/[E_G]$ the maximum scaled acclimation energy density to be achieved. Then, the rate of change of acclimation energy density

$$\frac{da}{dt} = \kappa_C r. \tag{S30}$$

Inserting (S29) into the equation above gives (9).

Differentiating between active and inactive cells

Even though only active cells contribute to growth of a population, many methods of estimating population sizes (including the optical density used in this paper) do not distinguish between active and inactive cells. This may present a problem during later stages of growth, when a large proportion of cells could be inactive. We therefore tried modeling active and inactive bacterial cells separately, but did not include this feature in the main text.

Active cells, denoted by V_A, acquired energy, used it for metabolism and cell division, and transformed into inactive cells, V_I, with the hazard rate, h. The hazard was, therefore, interpreted as probability of inactivation of active cells, not death. Once inactivated, cells lysed with the lysis rate, δ, which increased as a function of acquired aging acceleration. The aggregate measure of bacterial biomass used here (OD) then corresponded to the sum of both types of cells, $V_I + V_A$.

Separating active and inactive cells introduced significant complexity (new state variable, new parameters) and only marginally improved the fit by reducing the difference between the control and first two treatments. Simulated proportions of inactive cells in the model were very low (1-4%) because the lysis rate was high (0.30 per hour). We therefore decided to leave out the distinction between the two types of cells, while recognizing that it may be an important feature.

Distinction between active and inactive cells can be dynamically very important when proportion of inactive cells is significant. In a model where only active cells are considered, all cells divide, and all are subject to death and lysis. Calculating growth rate assuming all cells divide underestimates the division rate of individual cells (and related physiological parameters) because a proportion of divisions is attributed to cells that were dormant. Furthermore, if the lysis rate is high, cell concentration (and the corresponding optical density) can drop to zero in a very short time - an effect rarely observed. In a model with both types of cells, however, only inactive cells are subject to lysis, so even with a high lysis rate there could be a standing, viable stock for a prolonged period.

Independent data on cell activity could help quantify the actual effect of inactive cells, but one has to be careful when comparing the model's interpretation of an inactive cell to measurements of cell activity. Activity levels are typically done by staining, with low fluorescence interpreted as 'inactive'. The model, however, predicts that *most* of cells at the end of the simulation will be active, but barely so (barely able to keep up the maintenance) so most of what a model would consider as 'active' cells could be classified as 'inactive' by a bio-assay.

Effects of damage-inducing compounds

We noted in the Introduction that damage-inducing compounds (aging acceleration) may affect energetic processes in the cell by increasing maintenance and decreasing growth efficiency. Neither mechanism was incorporated in the model described in the main text, but both can easily be added. We investigated the extended model as well.

We assumed that aging acceleration affects maintenance (m), costs of growth (investment ratio, g), and energy conductance (v). Assuming a cell has maintenance m, investment ratio g, and conductance v in the absence of significant aging acceleration, the modified maintenance, m', investment ratio, g', and

conductance, v', are then (in terms of aging acceleration, q)

$$m' = me^{\gamma_m q}, \quad \text{(S31)}$$
$$g' = ge^{\gamma_g q}, \text{ and} \quad \text{(S32)}$$
$$v' = ve^{\gamma_v q}, \quad \text{(S33)}$$

where γ_m, γ_g and γ_v are the aging acceleration maintenance and energy investment ratio cost coefficients, and effects on v are multiplicative with (6) and (8).

We found that including effects of aging acceleration on maintenance and growth only marginally improved the fit, and did not warrant inclusion in the final version of the model without additional data enabling better characterization of the effects. Linear instead of exponential dependence produced similar results.

Experimental setup of the Cd-exposure experiment

P. aeruginosa PG201 (Urs Ochsner, University of Colorado) was cultured in Luria Bertani (LB) broth amended with varying concentrations (0, 5, 10, 20, 37.5, 75, 115, and 150 mg(total Cd)/L) of cadmium added as cadmium acetate ($Cd(CH_3COO)_2$). Prior to the experiments, archived bacteria (maintained at -80^oC in 70% LB/30% glycerol, v/v) for inocula were streaked onto LB agar and incubated for 18 hours at 30^oC. Culturing was under aerobic conditions in the dark (200 rpm, 30^oC) using a BioTek Synergy 2 microplate reader (Winooski, VT). Individual cultures were repeated in triplicate in volumes of 200 μL. Optical density at 600 nm (OD_{600}) was measured automatically during growth once every hour. Average initial OD ranged from 0.003 for the control to the maximum of 0.0066 for the treatments. All chemicals were reagent grade or better (Sigma Chemical, St. Louis, MO; and Fisher Scientific, Hampton, NH). Intracellular ROS was quantified at 15 hours for 0, 10, 20, 37.5, 75, and 125 mg(total Cd)/L using the 2',7'-dichlorofluorescein diacetate (DCFH-DA) assay, as described in Priester *et al.* [1]. ROS levels were expressed as H_2O_2 equivalents per liter.

Acknowledgments

References

1. Priester J, Stoimenov P, Mielke R, Webb S, Ehrhardt C, et al. (2009) Effects of soluble cadmium salts versus cdse quantum dots on the growth of planktonic *Pseudomonas aeruginosa*. Environmental Science & Technology 43: 2589-2594.

2. Kooijman S (2010) Dynamic Energy Budget theory for metabolic organisation, 3rd ed. Cambridge University Press, Great Britain. ISBN 9780521131919.

3. Nisbet R, Muller E, Lika K, Kooijman S (2000) From molecules to ecosystems through dynamic energy budget models. Journal of Animal Ecology 69: 913-926.

4. Sousa T, Domingos T, Kooijman S (2008) From empirical patterns to theory: a formal metabolic theory of life. Philosophical Transactions of the Royal Society B 363: 2453-2464.

5. Muller E, Nisbet R, Berkley H (2010) Sublethal toxicant effects with dynamic energy budget theory: model formulation. Ecotoxicology 19: 38-47.

6. Jager T, Vandenbrouck T, Baas J, de Coen W, Kooijman S (2010) A biology-based approach for mixture toxicity of multiple endpoints over the life cycle. Ecotoxicology 19: 351-361.

7. Hanegraaf P, Muller E (2001) The dynamics of the macromolecular composition of biomass. Journal of Theoretical Biology 212: 237-251.
